# Supplementary material for: Content-rich biological network constructed by mining PubMed abstracts
Source: BMC Bioinformatics. 2004 Oct 8;5:147. doi: 10.1186/1471-2105-5-147 (PMC528731; doi:10.1186/1471-2105-5-147)
Supplement: Additional File 2 — The original results of the above study (non-essential files are deleted to keep the file size under the limit set by BMC bioinformatics). [file 1471-2105-5-147-S2.bz2 › chilibotAdditionalFile2/dip10/274ID1922387E1094/html/MAPK9_JUN.html]

 


 **MAPK9** and **JUN** 
  
Found 316 abstracts in PubMed, retrieved 10.  
 

 What does Google say? 
 PDF only 
| .edu only 

---

**Interactive relationship** (e.g. stimulation, inhibition, etc)

**Non-interactive relationship** (e.g. studied together, co-existance, homology, etc.)

- These same peptides also inhibited JNK2  [ **MAPK9** ]  mediated phosphorylation of c  **Jun**  and ATF2, suggesting that transcription factors, MKK4, and the JIP scaffold compete for docking to JNK.  Ref: 12788955 J Biol Chem< MedlineTA>J Biol Chem,
- Thus, we suspected that either  **Jun**  N terminal kinase 2 JNK 2  [ **MAPK9** ]  or Src family proteins were involved in HLA DR mediated signaling.  Ref: 12842758 Cytokine, 2003
- By immunoblotting, these proteins were identified as c  **Jun**  N terminal kinase JNK 2  [ **MAPK9** ]  and p38 mitogen activated protein kinase MAPK delta, respectively, both of which are believed to be involved in apoptosis induction by CDDP.  Ref: 12824923 Cancer Sci< MedlineTA>Cancer Sci, 2003
- 5 dihydroxyvitamin D3 inhibits ultraviolet B induced apoptosis,  **Jun**  kinase  [ **MAPK9** ]  activation, and interleukin 6 production in primary human keratinocytes.  Ref: 12858333 J Cell BiochemJ Cell Biochem, 2003
- The activation of one of these, the  **Jun**  kinase  [ **MAPK9** ]  pathway, has been implicated in apoptotic responses to DNA damage, cell stress and cytotoxic drugs.  Ref: 12860462 Drug Resist Updat, 2003
- Role of  **Jun**  and  **Jun**  kinase  [ **MAPK9** ]  in resistance of cancer cells to therapy.  Ref: 12860462 Drug Resist Updat, 2003
- 3 Through activation of the  **Jun**  kinase  [ **MAPK9** ]  pathway AP 1  [ **JUN** ]  is induced, leading to cell cycle arrest in the G0 G1 phase.  Ref: 12790790 Endocr Relat Cancer, 2003
